# Supplementary material for: Effects of EOs vs. Antibiotics on E. coli Strains Isolated from Drinking Waters of Grazing Animals in the Upper Molise Region, Italy
Source: Molecules. 2022 Nov 24;27(23):8177. doi: 10.3390/molecules27238177 (PMC9741016; doi:10.3390/molecules27238177)
Supplement: Supplementary file 1 [file molecules-27-08177-s001.zip › molecules-2029894-supplementary.pdf]

molecules

Supplementary Materials

Effects of EOs vs. Antibiotics on *E. coli* Strains Isolated from Drinking Waters of Grazing Animals in the Upper Molise Region, Italy

Chiara Aquilano, Ligia Baccari, Claudio Caprari, Fabio Divino, Francesca Fantasma, Gabriella Saviano and Giancarlo Ranalli *

Department of Biosciences and Territory, University of Molise; C. da Fonte Lappone snc, 86090 Pesche, IS, Italy

* Correspondence: ranalli@unimol.it; Tel.: +39-874-404155

Table S1. Chemical composition of the commercial essential oil of *Cinnamomum verum.*

N.	Compound	Exp RI	Ref RI	Area % - SD	Abbr.
1	Benzaldeide	965	960	0.07 ± 0.01	OT
2	Cymene <*p*->	1026	1024	0.01 ± 0.00	MM
3	Limonene	1031	1029	1.35 ± 0.02	MM
4	Linalool	1101	1096	0.03 ± 0.01	AMO
5	Phenylethyl Alchool	1117	1107	0.04 ± 0.01	OT
6	Benzenepropanal	1166	1160	0.03 ± 0.01	MMO
7	Terpinen-4-ol	1179	1177	0.02 ± 0.00	MMO
8	Terpineol <*α*->	1192	1188	0.01 ± 0.00	MMO
9	Cinnamaldehyde <(Z)->	1222	1219	0.48 ± 0.09	MMO
10	Anisaldehyde <*o*->	1246	1242	0.05 ± 0.00	MMO
11	Cinnamaldedyde <(E)->	1275	1270	77.14 ± 0.30	MMO
12	Eugenol	1365	1359	18.76 ± 0.23	MMO
13	Thujic acid	1377	1382	0.06 ± 0.01	MMO
14	Coumarin	1440	1434	0.15 ± 0.01	OT
15	Cinnamyl acetate <(E)->	1449	1446	0.18 ± 0.01	MMO
16	Cadinene <δ->	1526	1523	0.03 ± 0.00	AS
17	Methoxy cinnamaldehyde <(E)-*o*->	1536	1528	1.36 ± 0.04	MMO
18	7-Methyl-1-naphthol	1570	1565	0.03 ± 0.01	OT
19	Spathulenol	1580	1578	0.02 ± 0.01	BSO
20	Caryophyllene oxide	1586	1583	0.02 ± 0.00	BSO
21	Amyl cinnamaldehyde <(Z)->	1650	1649	0.02 ± 0.00	MSO
22	Cadalene	1676	1676	0.01 ± 0.00	BS

Abbreviations: AM-aliphatic monoterpenes; MM-monocyclic monoterpenes; BM-bi-and tricyclic monoterpenes; AMO- aliphatic monoterpenoids; MMO- monocyclic monoterpenoids; BMO- bi-and tricyclic monoterpenoids; AS- aliphatic sesquiterpenes; MS- monocyclic sesquiterpenes; BS- bi- and tricyclic sesquiterpenes; ASO- aliphatic sesquiterpenoids; MSO- monocyclic sesquiterpenoids; BSO- bi- and tricyclic sesquiterpenoids, OT- others. SD- standard deviation; Exp. RI- experimental retention index; Ref. RI- literature data.

Table S2. Chemical composition of commercial essential oil of *Timus vulgaris.*

N.	Compound	Exp RI	Ref RI	Area % - SD	Abbr.
1	Thujene <α->	931	930	0.04 ± 0.01	MM
2	Pinene <α->	937	939	1.33 ± 0.02	MM
3	Fenchene <α->	951	952	0.47 ± 0.03	MM
4	Pinene <β->	978	979	0.16 ± 0.02	MM
5	Myrcene	994	990	0.59 ± 0.02	AM
6	Phellandrene <α->	1004	1002	0.07 ± 0.02	MM
7	Terpinene <α->	1019	1017	0.58 ± 0.01	MM
8	Cymene <p->	1030	1024	21.83 ± 1.16	MM
9	Limonene	1034	1029	22.12 ± 1.53	MM
10	Terpinene <γ->	1062	1059	0.25 ± 0.01	MM
11	Terpinolene	1089	1088	0.20 ± 0.01	MM
12	Linalool	1103	1096	2.47 ± 0.02	AMO
13	Camphor	1147	1146	0.18 ± 0.02	BMO
14	Isoborneol	1159	1160	0.18 ± 0.01	MMO
15	borneol	1168	1169	0.42 ± 0.03	MMO
16	Terpinen-4-ol	1179	1177	0.07 ± 0.02	MMO
17	Terpineol <α->	1191	1188	0.35 ± 0.19	MMO
18	Terpineol <γ->	1195	1199	0.71 ± 0.19	MMO
19	Thymol	1297	1290	38.05 ± 0.17	MMO
20	Carvacrol	1309	1299	8.70 ± 0.14	MMO
21	Caryophyllene <(E)->	1420	1419	0.78 ± 0.02	BS
22	Humulene <α->	1455	1454	0.09 ± 0.01	MS
23	Cadinene <α->	1526	1523	0.02 ± 0.0	BS
24	Caryophyllene oxide	1585	1583	0.13 ± 0.02	BSO

Abbreviations: AM-aliphatic monoterpenes; MM-monocyclic monoterpenes; BM-bi-and tricyclic monoterpenes; AMO- aliphatic monoterpenoids; MMO- monocyclic monoterpenoids; BMO- bi-and tricyclic monoterpenoids; AS- aliphatic sesquiterpenes; MS- monocyclic sesquiterpenes; BS- bi- and tricyclic sesquiterpenes; ASO- aliphatic sesquiterpenoids; MSO- monocyclic sesquiterpenoids; BSO- bi- and tricyclic sesquiterpenoids, OT- others. SD- standard deviation; Exp. RI- experimental retention index; Ref. RI- literature data.

Table S3. Chemical composition of commercial essential oil of *Melaleuca alternifolia.*

N.	Compound	Exp RI	Ref RI	Area % - SD	Abbr.
1	Pinene <α->	937	939	11.52 ± 0.38	BM
2	Fenchene <α->	950	952	0.27 ± 0.03	BM
3	Pinene <β->	977	979	4.24 ± 0.16	BM
4	Phellandrene <α->	1002	1002	0.16 ± 0.02	MM
5	Terpinene <α->	1018	1017	8.15± 0.22	MM
6	Cymene <p->	1027	1024	5.01 ± 0.23	MM
7	Limonene	1031	1029	2.25 ± 0.35	MM
8	Cineole <1.8->	1034	1031	3.84 ± 0.31	BMO
9	Terpinene <γ->	1063	1059	16.52 ± 0.46	MM
10	Terpinolene	1089	1088	2.50 ± 0.13	MM
11	Terpinen-4-ol	1180	1177	32.52 ± 0.64	MMO
12	Terpineol <α->	1193	1188	4.98 ± 0.08	MMO
13	Methyl chavicol	1199	1196	0.68 ± 0.01	MMO
14	Thymol	1295	1290	0.06 ± 0.05	MMO
15	Carvacrol	1303	1299	0.03 ± 0.02	MMO
16	Gurjunene <α->	1410	1409	0.26 ± 0.01	BS
17	Caryophyllene <(E)->	1420	1419	0.04 ± 0.01	BSO
18	Guaiene <α->	1440	1439	3.24 ± 0.05	BS
19	Aromadendrene <allo->	1462	1460	0.12 ± 0.01	BS
20	Selinene <β->	1487	1490	0.24 ± 0.00	BS
21	Selinene <δ->	1489	1492	0.29 ± 0.02	BS
22	Valencene	1496	1496	1.79 ± 0.01	BS
23	Muurolene <α->	1501	1500	0.09 ± 0.02	BS
24	Globulol	1586	1590	0.40 ± 0.01	BSO

Abbreviations: AM-aliphatic monoterpenes; MM-monocyclic monoterpenes; BM-bi-and tricyclic monoterpenes; AMO- aliphatic monoterpenoids; MMO- monocyclic monoterpenoids; BMO- bi-and tricyclic monoterpenoids; AS- aliphatic sesquiterpenes; MS- monocyclic sesquiterpenes; BS- bi- and tricyclic sesquiterpenes; ASO- aliphatic sesquiterpenoids; MSO- monocyclic sesquiterpenoids; BSO- bi- and tricyclic sesquiterpenoids, OT- others. SD- standard deviation; Exp. RI- experimental retention index; Ref. RI- literature data.
